# Statistical regularities cause attentional suppression with target-matching distractors

**DOI:** 10.3758/s13414-020-02206-9

**Published:** 2020-11-29

**Authors:** Dirk Kerzel, Stanislas Huynh Cong

**Affiliations:** Faculté de Psychologie et des Sciences de l’Education, Université de Genève, 40 Boulevard du Pont d’Arve, 1205 Genève, Switzerland

**Keywords:** Visual search, Attentional capture, Statistical learning, Attentional suppression

## Abstract

Visual search may be disrupted by the presentation of salient, but irrelevant stimuli. To reduce the impact of salient distractors, attention may suppress their processing below baseline level. While there are many studies on the attentional suppression of distractors with features distinct from the target (e.g., a color distractor with a shape target), there is little and inconsistent evidence for attentional suppression with distractors sharing the target feature. In this study, distractor and target were temporally separated in a cue–target paradigm, where the cue was shown briefly before the target display. With target-matching cues, RTs were shorter when the cue appeared at the target location (valid cues) compared with when it appeared at a nontarget location (invalid cues). To induce attentional suppression, we presented the cue more frequently at one out of four possible target positions. We found that invalid cues appearing at the high-frequency cue position produced less interference than invalid cues appearing at a low-frequency cue position. Crucially, target processing was also impaired at the high-frequency cue position, providing strong evidence for attentional suppression of the cued location. Overall, attentional suppression of the frequent distractor location could be established through feature-based attention, suggesting that feature-based attention may guide attentional suppression just as it guides attentional enhancement.

The visual system is confronted with more sensory information than it can process. Selective attention is thought to reduce the amount of visual information by filtering out sensory signals that are irrelevant for the task at hand (Bundesen, Habekost, & Kyllingsbaek, 2005; Desimone & Duncan, 1995; Schneider, 2013; Tsotsos, Kotseruba, Rasouli, & Solbach, 2018). To locate relevant information, the incoming sensory information is matched to a stored representation of the target features, which is referred to as attentional template (Duncan & Humphreys, 1989), target template (Vickery, King, & Jiang, 2005), or attentional control set (Folk, Remington, & Johnston, 1992). Attentional templates may contribute to several stages of visual search. Initially, attentional templates may enhance the target features in a spatially global manner by activating feature-based attention (Andersen, Hillyard, & Muller, 2013; Maunsell & Treue, 2006; W. Zhang & Luck, 2009). Then, feature-based attention is thought to guide location-based attention to the target location (Eimer, 2014; Wolfe, 2007), where it enhances stimulus processing. For instance, perceptual sensitivity improves (Carrasco, 2011) and reaction times (RTs) decrease (Chica, Martin-Arevalo, Botta, & Lupianez, 2014).

To investigate the nature of attentional templates, the contingent capture paradigm by Folk et al. (1992) has proven useful. The initial assumption was that the attentional template stored in memory corresponds to the physical target features. For instance, the attentional template would correspond to “red” when participants are asked to search for a red target among white nontargets. Cues that were flashed briefly before the target display were used to demonstrate that the attentional template constrained attentional selectivity. Notably, only cues that matched the target properties captured attention. For instance, a red cue would capture attention when observers searched for a red target, but not when they searched for a green target (Folk & Remington, 1998). Attentional capture resulted in shorter RTs when the cue appeared at the same location as the target (valid cue) compared with when it appeared at a different location (invalid cue).

While the contingent capture paradigm has been frequently used to study characteristics of the attentional template (e.g., Ansorge & Becker, 2014; Becker, 2010; Folk & Remington, 1998; Harris, Jacoby, Remington, Travis, & Mattingley, 2019; Kerzel, 2019, 2020; Schönhammer, Grubert, Kerzel, & Becker, 2016), less is known about the ability of attentional templates to guide the deployment of attentional suppression. The role of attention in the contingent capture paradigm was mostly limited to the enhanced processing at the cued position. Consistent with enhancement, RTs on valid trials were not only shorter than RTs on invalid trials, but also shorter than RTs on neutral trials without a cue (Burnham, 2019; Folk & Remington, 1998; Ruthruff & Gaspelin, 2018; Schönhammer, Becker, & Kerzel, 2020). While the enhanced processing at the cued location is a basic tenet of the contingent capture paradigm, some recent studies investigated whether there is also attentional suppression (Burnham, 2018; Leber, Gwinn, Hong, & O’Toole, 2016; Ruthruff & Gaspelin, 2018; Schönhammer et al., 2020). Attentional suppression is thought to reduce the impact of salient, but irrelevant distractors (Gaspelin & Luck, 2018b; Geng, 2014; Liesefeld & Müller, 2019) and has been mostly studied in the additional singleton paradigm (Theeuwes, 2018, 2019). Target and distractor features in the additional singleton paradigm must be distinct because they are presented simultaneously. In a typical variant of the additional singleton paradigm, the target is defined by its shape and on some trials, a distractor with a salient color is shown. RTs are generally longer on distractor-present than distractor-absent trials. However, when the search goals are sufficiently precise, the salient-but-irrelevant distractor may be suppressed, which reduces the delay of RT caused by the distractor (Gaspelin, Leonard, & Luck, 2015, 2017; Gaspelin & Luck, 2018a; but see Kerzel & Burra, 2020; Wang & Theeuwes, 2020). To conclusively demonstrate that the distractor was suppressed and not just ignored, performance was compared with a baseline condition. For instance, Gaspelin et al. (2015) compared letter identification at the location of the salient distractor to the location of an inconspicuous nontarget element. Performance was worse at the distractor location than at the baseline location, suggesting that the distractor was suppressed, and not just ignored (see also Chang & Egeth, 2019).

Some evidence for attentional suppression in the contingent capture paradigm comes from “same location costs” where RTs are longer with valid than invalid cues, which is the opposite of the typical enhancement with valid cues. Same location costs have been reported for cues that do not match the target, in combination with heterogeneous search displays (Carmel & Lamy, 2014; Eimer, Kiss, Press, & Sauter, 2009; Kerzel, 2019; Lamy & Egeth, 2003; Schoeberl, Ditye, & Ansorge, 2018). However, the reasons for the inverted cueing effects are disputed with some studies pointing to object updating costs (Carmel & Lamy, 2014, 2015; but see Schoeberl et al., 2018) and others favoring attentional suppression (Kerzel, 2019). A recent study using event-related potentials did not provide evidence for attentional suppression because an electrophysiological marker of attentional suppression, the P_D_ component (Hickey, Di Lollo, & McDonald, 2009), was absent (Schönhammer et al., 2020). In addition, RTs did not differ from neutral trials, suggesting that performance was not below baseline as would be expected if attentional suppression had occurred.

While attentional suppression may not account for same location costs with valid cues that do not match the target, there are some studies suggesting that attentional suppression may reduce the cost of invalid cues that match the target. The typical finding with invalid target-matching cues is that RTs increase relative to trials without cues or with neutral cues (Burnham, 2019; Folk & Remington, 1998; Schönhammer et al., 2020), indicating that invalid target-matching cues disrupt visual search. The increase of RTs may be attenuated when the cue appears at a location that is attentionally suppressed. In other words, attentional suppression is thought to prevent attentional capture by invalid target-matching cues. However, previous studies testing this hypothesis have yielded inconsistent results. In the first study on this topic, Leber et al. (2016) combined the contingent capture paradigm with an endogenous cueing procedure. Between 300 and 650 ms before the cue–target displays, a central arrow indicated where the target was most likely to occur. Because attention was endogenously shifted in the direction of the arrow, RTs decreased for targets in the corresponding direction (see Posner, 1980). More interestingly, each arrow direction was associated with a location where the target-matching cue was most likely to occur. Although participants were unaware of the association between arrow direction and frequent cue location, disruption by invalid cues was attenuated when they were presented on the frequent cue location. Leber et al. (2016) concluded that implicit learning allowed participants to suppress locations where salient but irrelevant stimuli are expected to occur. However, there was no baseline condition in the RT task, and it is therefore unclear whether invalid cues were suppressed or just successfully ignored.^1^ Clear evidence for attentional suppression would require performance different from baseline.

Further, the results of a recent study do not substantiate the conclusion that attentional suppression reduces the impact of invalid target-matching cues. In the study by Burnham (2018), an arrow cue was presented for 1,500 ms to indicate where the target in the following cue–target display would not occur. Participants were instructed to ignore this location. RTs decreased with increasing distance between the to-be-ignored location and the target, showing that participants successfully ignored the indicated location. However, capture by invalid cues on the ignored location was not reduced compared with invalid cues on other locations, suggesting that attentional capture occurred even on ignored locations. However, Ruthruff and Gaspelin (2018) reported a conflicting result, which may result from their different experimental design. In Ruthruff and Gaspelin (2018), the to-be-ignored locations were fixed across trials whereas they changed from trial to trial in Burnham (2018). That is, the target in Ruthruff and Gaspelin (2018) could appear on only two out of four locations and participants were encouraged to ignore the irrelevant locations from the start. In addition, target-matching foils were presented on the irrelevant locations to force participants to ignore these locations. Invalid cues on the ignored locations captured attention less than invalid cues on the attended locations. However, performance did not differ from baseline. Several baseline conditions were tested, such as conditions without cues or with central cues, and conditions with cues in a nonmatching color. In all experiments, RTs with invalid cues on the ignored locations were never different from RTs in the baseline conditions, providing no evidence for attentional suppression of to-be-ignored locations. Overall, one of the reviewed studies concluded in favor of attentional suppression (Leber et al., 2016), whereas two others found no evidence for attentional suppression (Burnham, 2018; Ruthruff & Gaspelin, 2018), but some evidence for the ability to ignore invalid cues at irrelevant locations (Ruthruff & Gaspelin, 2018).

Because of these empirical inconsistencies, the primary goal of the present study was to provide additional evidence for attentional suppression in a cueing paradigm. We opted for a procedure that induced attentional suppression based on trial history (Awh, Belopolsky, & Theeuwes, 2012; Theeuwes, 2019). Following previous research on the additional singleton paradigm (see General Discussion), we used statistical regularities of cue locations to promote attentional suppression. In contrast to studies on the additional singleton paradigm, however, we evaluated whether suppression may occur for cues sharing a task-relevant, and therefore attended, feature. Thus, resolving the empirical inconsistencies surrounding attentional suppression in cueing paradigms allows for new theoretical insights into the interplay between feature-based attention and attentional suppression. Typically, the assumed role of feature-based attention is to guide attentional enhancement (Eimer, 2014; Wolfe, 2007). Here, we tested whether feature-based attention may also guide attentional suppression.

In a classic study on statistical regularities, Reder, Weber, Shang, and Vanyukov (2003) presented a distractor more frequently at one out of four possible positions while the target appeared with equal frequency at all possible positions. Reder et al. (2003) noted two effects. First, interference from the irrelevant distractor was reduced at the frequent-distractor position compared with the other locations. Second, target processing was impaired when the target appeared on the frequent-distractor location compared with a position where the distractor was never shown. Subsequent research attributed the reduced distractor interference to the shielding of search from likely distractor positions (e.g., Goschy, Bakos, Müller, & Zehetleitner, 2014), altered distractor filtering (e.g., Ferrante et al., 2018), or attentional suppression (e.g., Wang & Theeuwes, 2018b).

To provide evidence for attentional suppression in the contingent capture paradigm, we looked for a pattern of results that resembled Reder et al. (2003). We expect the effect of invalid cues to be attenuated on locations where the cue is frequently presented. This effect represents a conceptual replication of Leber et al. (2016), but with a fixed location. In addition, we expect processing of the target to be impaired on this location. Importantly, impaired target processing is not predicted when only participants’ ability to ignore the frequent cue event improves. The reason is that cue and target events are temporally separated and different by their form (see Fig. 1). Thus, improving the ability to ignore the cue is not expected to affect target processing. In contrast, attentional suppression of the frequent cue location predicts not only reduced capture by invalid cues, but also impaired processing of any other stimulus on this location. Thus, we looked for impaired target processing to provide additional evidence for attentional suppression in the contingent capture paradigm. Another possibility would be to run a neutral condition to show that RTs with invalid cues on the suppressed location are different from baseline. However, comparisons with neutral conditions are difficult to interpret, as there are many ways to design a neutral condition. For instance, neutral conditions typically omit the cue stimulus or present a supposedly neutral cue, but both solutions may cause spurious differences (Schönhammer et al., 2020; see also Jonides & Mack, 1984). Therefore, impaired target processing may provide more conclusive evidence in favor of attentional suppression.

**Fig. 1:**
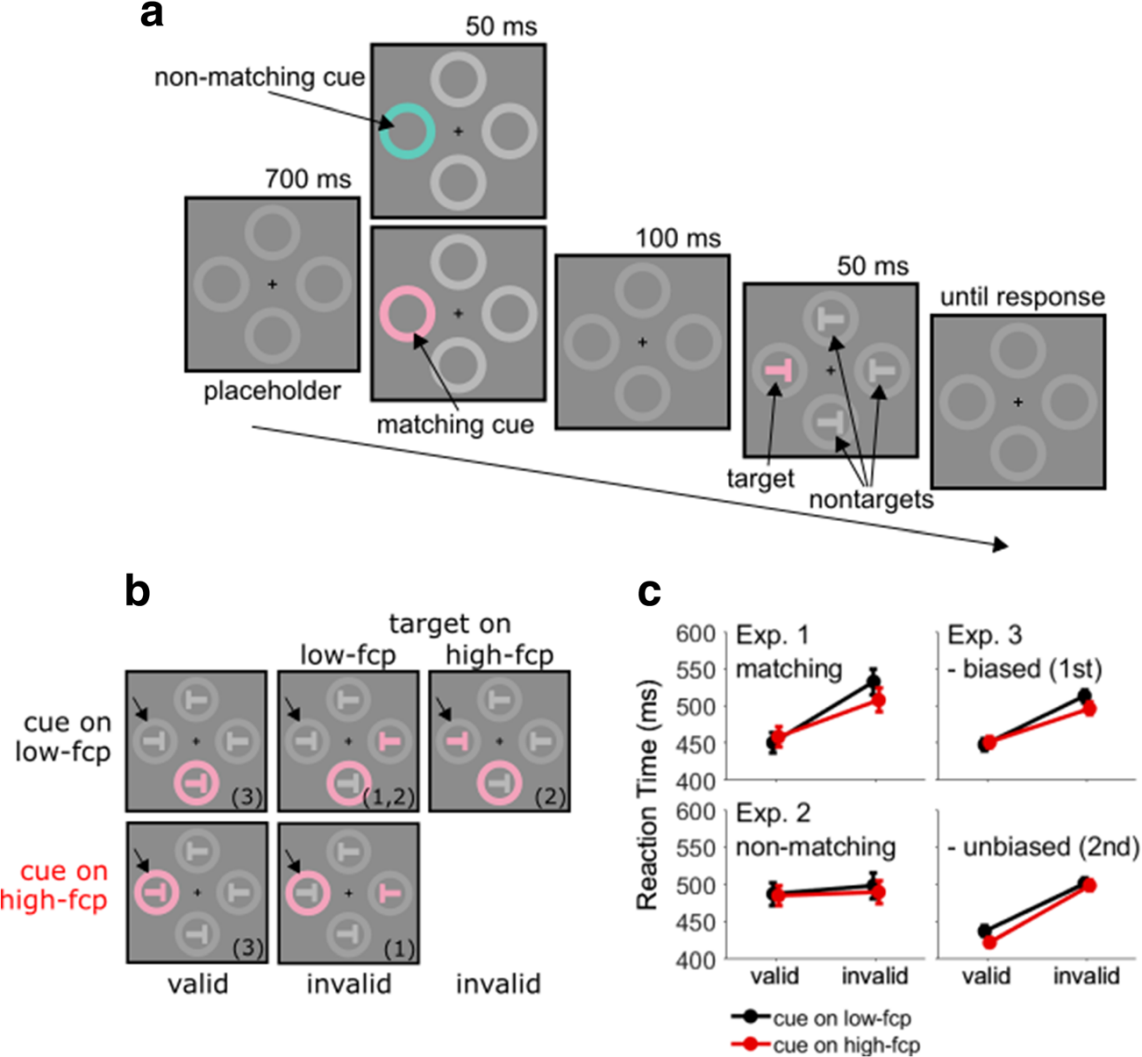
**a** Experimental stimuli (not drawn to scale, placeholders are simplified) and the time course of a trial. A cue display was shown briefly before the target display. The cue was in the target color (matching cue, Experiments 1 and 3) or in a different color (nonmatching cue, Experiment 2). **b** Experimental conditions by superposing cue and target displays. The high-frequency cue position (high-fcp) is indicated by an arrow. The three remaining positions are low-frequency cue positions (low-fcp). The comparisons of interest are indicated in parentheses (see also Table 1). **b** Results as a function of frequency of cue position and cue validity. For invalid cues on low-frequency cue positions, the data were collapsed across targets on low-fcp and high-fcp

## Experiment 1

In Experiment 1, the cue appeared on the high-frequency cue position in 70% of trials and on one of three low-frequency cue positions in 30% of trials. Cue and target positions were independent, resulting in 25% valid and 75% invalid cues. Figure 1b and Table 1 show the distribution of trials as a function of cue validity, cue position, and target position. Attentional suppression of the high-frequency cue position may have two effects: reduced capture from invalid cues and impaired processing of targets. These two processes were teased apart in two comparisons. The first comparison isolates the reduced capture of invalid cues by restricting the analysis to *targets* on low-frequency cue positions (see column 2 in Fig. 1b and lines 1–2 in Table 1). If there was attentional suppression of the high-frequency cue position, invalid cues on the high-frequency cue position are expected to capture attention less than invalid cues on a low-frequency cue position. Therefore, the delay in RTs should be reduced for invalid cues on the high-frequency cue position. The second comparison isolates impaired target processing by restricting the analysis to invalid *cues* on a low-frequency cue position (see columns 2–3 in Fig. 1b and lines 2–3 in Table 1). If there was impaired target processing resulting from attentional suppression of the high-frequency cue position, RTs are expected to be delayed for targets on the high-frequency compared with the low-frequency cue positions. Another possible comparison concerned valid trials (see column 1 in Fig. 1b and lines 4–5 in Table 1), which confounds cue-related and target-related processes because cue and target were presented on the same position. As we do not have predictions about how these processes may interact, we ran this comparison in an explorative manner.

**Table 1:** The number of trials as a function of cue validity, cue position, and target position in Experiments 1 and 2

	Cue	Target	# Trial	Comparison
Invalid	high-fcp	low-fcp	420	1
	low-fcp	low-fcp	120	1, 2
	low-fcp	high-fcp	60	2
Valid	high-fcp	high-fcp	140	3
	low-fcp	low-fcp	60	3

*Note.* The total number of trials was 800. The positions where the cue occurred on 70% and 30% of trials are referred to as high-frequency cue position (high-fcp) and low-frequency cue position (low-fcp), respectively. The three comparisons of theoretical interest are indicated in the last column. The first isolates cue-related processing. The second isolates target-related processing. The third comparison confounds cue and target-related processing

### Methods

#### Participants

In a previous study, we found the effect size for the difference in cueing effects between target-matching and target-nonmatching colors to be about Cohen’s *d*_*z*_ = 1.4 (Kerzel, 2020). When aiming for a power of 0.8 with a Type 1 error rate of 5%, the necessary sample size is 7. We think that the difference between cueing effects for frequent and infrequent cues may be on the same order, but we cannot know for sure. Therefore, we increased the sample size to 12, which allowed us to detect effects with a Cohen’s *d*_*z*_ as low as 0.89. Twelve undergraduate psychology students participated in Experiment 1 (one male; age: *M* = 21.9 years, *SD* = 4.9) and another 12 in Experiment 2 (two males; age: *M* = 20.1 years, *SD* = 1.9). Because fewer trials were run per condition in Experiment 3, we increased the sample size to 16 (one male; age: *M* = 20.4 years, *SD* = 2.1), which allowed us to detect effect sizes as low as 0.75. Psychology students participated for class credit and reported normal or corrected-to-normal vision. The study was approved by the ethics committee of the Faculty of Psychology and Educational Sciences and was carried out in accordance with the Code of Ethics of the World Medical Association (Declaration of Helsinki). Informed consent was given before the experiment started.

#### Apparatus

Stimuli were displayed on a 22.5-inch LCD monitor at 100 Hz with a resolution of 1,920 × 1,200 pixels (VIEWPixx Light, VPixx Technologies Inc., Saint-Bruno, Canada), driven by an AMD Radeon HD 7470 graphics card with a color resolution of 8 bits per channel. CIE1931 chromaticity coordinates and luminance (xyY with Y in cd/m^2^) of the monitor primaries were R = (0.672, 0.312, 53.2), G = (0.091, 0.75, 123.4), and B = (0.1, 0.094, 20.5). The white-point of CIELAB-space was xyY = (0.274, 0.356, 194.6). Gamma corrections were applied based on the measured gamma curves of the monitor primaries. Colors were measured with a Cambridge Research Systems (Rochester, Kent, UK) ColorCAL MKII colorimeter. Head position was stabilized with a chin and forehead rest at a viewing distance of 66 cm.

#### Stimuli

There was a placeholder, a cue, and a target display. The placeholder display was composed of a central fixation cross (0.2° radius, 0.07° line width) and four outline rings, all drawn in light gray. The distance from the center of the fixation cross to the center of the outline rings was 3°. The inner and outer rim of the outline rings corresponded to two circles with a radius of 1.1° and 1.4°, respectively. The line width was 1 pixel or 0.02°. In the cue display, all rings were filled. Three rings were filled with the same light gray as the circles and one ring with a color. In the target display, the letter *T* rotated by 90° clockwise or counterclockwise was shown in each placeholder. The bars making up the rotated *T* were 1° long and 0.2° thick. The target *T* was colored while the three nontarget *T*s were achromatic. The cue color was the same as the target color.

Stimuli were presented on an achromatic background with the chromaticities of the white-point and a lightness of L* = 45, which corresponds to a luminance of 29.2 cd/m^2^. The placeholders, the achromatic cues and nontarget *T*s were light gray (L* = 61 or 58.7 cd/m^2^). The colors that served as cue and target colors were sampled along an isoluminant hue circle at a lightness of L* = 61 with a saturation of 59. We selected four colors at angles of 0°, 90°, 180°, and 270°, which correspond to rose, amber, turquoise, and violet. The isoluminant colors in CIELAB-space (Fairchild, 2005; Witzel & Gegenfurtner, 2015, 2018) were used for consistency with our prior research (e.g., Huynh Cong & Kerzel, 2020; Kerzel, 2019; Kerzel & Witzel, 2019), but we do not think it would make a difference if other highly discriminable colors were used.

#### Design

The frequency of cue presentation on the four possible positions was biased. Cue presentation occurred on the high-frequency position on 70% of trials, and on 10% of trials on each of the three low-frequency positions. High-frequency and low-frequency cue positions were equally likely to be followed by any of the four target positions. That is, each cue was followed by a target on the same position (valid cues) on 25% of trials, and by a target on a different position (invalid cues) on 75% of trials. There were two blocks of 400 trials for a total of 800 trials. The high-frequency cue position (left, right, top, bottom) was fixed for each participant, but counterbalanced across participants. Target color was varied across participants. There were four participants each with a rose, amber, turquoise, and violet target.

#### Procedure

A trial started with the presentation of the unfilled placeholder rings. After 700 ms, the cue stimuli were shown for 50 ms, followed by the unfilled placeholders for 100 ms and the target stimuli for 50 ms. The resulting cue–target SOA was 150 ms. After target offset, the unfilled placeholders remained visible until a response was registered.

Participants responded to the orientation of the target *T* by clicking the corresponding mouse button (*T* rotated counterclockwise: left button, *T* rotated clockwise: right button). They were instructed to respond as rapidly and accurately as possible while ignoring the cue display.

Participants started the experiment by practicing the task until they felt comfortable with it. On average, participants performed 48 (*SD* = 32), 32 (*SD* = 12), and 30 (*SD* = 15) practice trials in Experiments 1–3, respectively. Visual feedback informed participants about choice errors, anticipations (RTs <0.2 s, which were extremely rare and will not be reported) and late trials (RTs >1.5 s). Every 100 trials, visual feedback about the percentage of correct responses and the median RTs were displayed during a self-terminated pause of at least 5 s.

#### Explicit learning assessment

At the end of the experiment, we asked observers to indicate the location where the cue had been presented more frequently. Some participants chose not to answer. In none of the experiments did a binomial test indicate that the proportion of participants indicating the correct position exceeded chance (*p*s > .28). The results are presented in Table 2.

**Table 2:** Results of the explicit learning assessment in Experiments 1–3

	*N*	Correct	Missing
Exp. 1	12	4	2
Exp. 2	12	4	1
Exp. 3	16	4	0

*Note.* In all experiments, the number of participants with a correct response was not significantly greater than the number expected by chance (binomial test). Participants who did not wish to respond were counted as missing

### Results

The data from all experiments are available in the Open Science Framework (https://osf.io/pe43x/). The following trials were removed from analysis of RTs. Trials with RTs outside the response window of 1.5 s (0.1%, 0.1%, 0.1% for Experiments 1–3), trials with choice errors (4.7%, 4.6%, 4.6% for Experiments 1–3), and trials with RTs that were two standard deviations above the respective condition mean (4.7%, 4.7%, 3.9% for Experiments 1–3). Significance was evaluated after correcting the false discovery rate (Benjamini & Hochberg, 1995), but uncorrected *p* values are reported.

As a manipulation check, we calculated cueing effects (invalid − valid) separately for cues on low-frequency and high-frequency cue positions, but collapsed across target positions (see Fig. 1c). The cueing effect was reduced for high-frequency compared with low-frequency cue positions (50 vs. 92 ms), *t*(11) = 5.08, *p* < .001, Cohen’s *d*_*z*_ = 1.47. Both cueing effects were significantly different from zero, *t*s(11) > 6.71, *p*s < .001, Cohen’s *d*_*z*_ > 1.93.

**Fig. 2:**
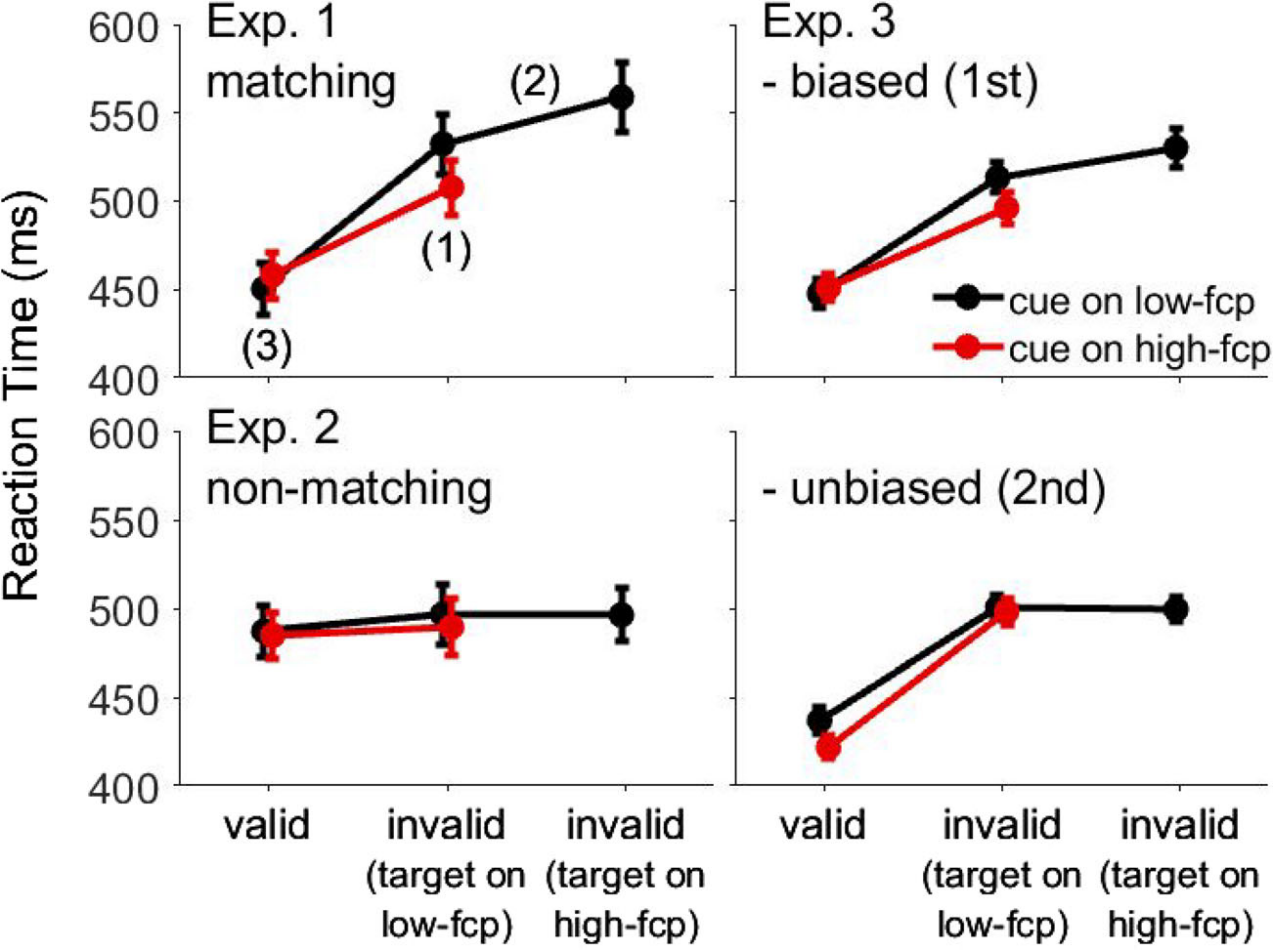
Experimental results from Experiments 1-3. Mean reaction times (RTs) are shown as a function of cue validity (valid, invalid), cue position (low-frequency cue position = “low-fcp”; high-frequency cue position = “high-fcp”), and target position (low-fcp, high-fcp). In Experiment 1, the cue was always matching whereas it was always nonmatching in Experiment 2. In Experiment 3, the cue was always matching, but the frequency of cue positions was only biased in the first block of trials. Error bars show the between-subject standard error of the mean

Next, we performed the two comparisons of interest. The respective means are shown in Fig. 2. First, we compared invalid cues on high-frequency and low-frequency cue positions, while the target was shown on a low-frequency cue position. The delay incurred by invalid cues was reduced when the cue was on the high-frequency cue position compared with when it was on the low-frequency cue position (508 vs. 533 ms), *t*(11) = 4.88, *p* < .001, Cohen’s *d*_*z*_ = 1.41, which suggests that interference was reduced as a result of attentional suppression of the high-frequency cue position. Second, we compared targets on high-frequency and low-frequency cue positions while the invalid cue was shown on a low-frequency cue position. RTs were delayed when the target was presented on the high-frequency compared with the low-frequency cue positions (562 vs. 533 ms), *t*(11) = 4.88, *p* < .001, Cohen’s *d*_*z*_ = 1.21, suggesting that target processing at the high-frequency cue location was impaired. Finally, we compared valid trials in an explorative manner, but found no difference between high-frequency and low-frequency cue positions (458 vs. 450 ms), *p* = .223. The same comparisons were also performed on error rates, but no significant results were observed, *p*s > .211.

Finally, we analyzed intertrial effects. Because the cue was more frequently presented at the high-frequency cue location, cue repetitions were more likely at this location. The cue may be considered a distractor. Effects of distractor repetition have garnered less attention than effects of target repetition. In general, repetition of target position and target color facilitate performance for 5–8 trials (Maljkovic & Nakayama, 1994, 1996). Similarly, the more frequent repetition of one out of several irrelevant target features facilitates target processing, but these effects subside rapidly when repetitions are balanced between all irrelevant target features (Jiang, Sha, & Remington, 2015; Kruijne & Meeter, 2015; Sha, Remington, & Jiang, 2017). While previous research noted short-term or long-term facilitation by repetition of target features, we find that presenting the distracting cue more frequently at one position resulted in attentional suppression. To test whether cue repetitions at the high-frequency cue position explained attentional suppression, we removed trials where the cue appeared at the same position as in the preceding trial. However, results were unchanged compared with the analysis of the full data set: The delay incurred by invalid cues was reduced when the cue was on the high-frequency cue position compared with when it was on the low-frequency cue position (503 vs. 535 ms), *t*(11) = 4.23, *p* = .001, Cohen’s *d*_*z*_ = 1.2, and RTs were delayed when the target was presented on the high-frequency compared with the low-frequency cue positions (559 vs. 535 ms), *t*(11) = 3.56, *p* = .005, Cohen’s *d*_*z*_ = 1.

### Discussion

We demonstrated that statistical regularities attenuate interference from a target-matching cue on the frequent cue location. At the same time, target processing was impaired at this position. Both effects suggest that there was attentional suppression of the frequent cue position. Further, suppression was not caused by immediate repetitions of the cue position because results did not change when we focused on trials where the cue position had changed.

## Experiment 2

Experiment 2 was a control experiment with a cue color that did not match the target color to show that suppression of the frequent distractor location depended on feature-based attention. It is known that nonmatching cue colors do not capture attention (e.g., Ansorge & Becker, 2014; Folk & Remington, 1998; Harris et al., 2019; Kerzel, 2019), suggesting that feature-based attention is not engaged.

### Methods

The methods were as in Experiment 1, with the exception that the cue color was different form the target color. The cue color was separated by 180° in CIELAB-space from the target color. For instance, a 0° target (rose) would be preceded by a 180° cue (turquoise, see Fig. 1a).

### Results

The overall cueing effects were not significant (see Fig. 1c), *p*s > .063. Further, none of the three comparisons of interest showed significant results (see Fig. 2), neither in RTs, *p*s > .089, nor choice error rates, *p*s > .454.

### Discussion

Experiment 2 showed that statistical regularities regarding cue position did not affect responses when the cue did not match the target feature. The absence of cueing effects suggests that attentional suppression did not result from the presentation of the salient cue event per se. Rather, suppression of frequent cue positions only occurred when the cue captured attention because it shared the task-relevant feature. It is therefore feature-based attention that guides location-based suppression. These findings complement previous research stressing the role of feature-based attention in guiding location-based enhancement (Eimer, 2014; Wolfe, 2007).

## Experiment 3

Some studies on the additional singleton paradigm evaluated whether the effect of statistical regularities carried over to a test block with balanced probabilities. Ferrante et al. (2018) asked participants to search for a shape-defined target and found that suppression of a location where a color-defined distractor appeared frequently did not persist in a test block with unbiased positional probabilities. In contrast, the frequency effect carried over to a test block (even after a 24-h pause) when target and distractor were drawn from the same perceptual dimension (i.e., orientation; Sauter, Liesefeld, & Müller, 2019). In the present paradigm, cue and target were not only drawn from the same perceptual dimension (i.e., color) but they also shared the same feature. Therefore, one may predict carryover from a block of trials with biased frequencies to a block with unbiased frequencies.

### Methods

The methods were as in the preceding experiments, with the following exception. The cue color was matching, and each participant worked through 960 trials. In the first block of 480 trials, the cue frequencies were biased as in Experiments 1 and 2. That is, the cue was shown in 70% of trials on the high-frequency position and in 30% on one of the remaining three positions. In the second block of 480 trials, the cue frequencies per position were unbiased. To evaluate whether differences between low-frequency and high-frequency positions persisted in the unbiased block, position labels (“low-frequency” and “high-frequency”) were carried over from the biased block. That is, the positions in the second block were analyzed according to the biased frequency distribution in the first block, even though frequencies were balanced. There were four participants each with a rose, amber, turquoise, and violet target.

### Results

Figure 1c shows that the overall cueing effect was reduced for high-frequency compared with low-frequency cue positions when the frequencies were biased (45 vs. 71 ms), *t*(15) = 5.09, *p* < .001, Cohen’s *d*_*z*_ = 1.27, whereas the opposite was the case when frequencies were unbiased (77 vs. 64 ms), *t*(15) = 2.14, *p* = .05, Cohen’s *d*_*z*_ = 0.53. All cueing effects were significantly different from zero, *t*s(15) > 8.12, *p*s < .001, Cohen’s *d*_*z*_ > 2.03.

Next, we performed the two comparisons of interest, separately for biased and unbiased blocks (see Fig. 2). Analyses for the biased block confirmed the results from Experiment 1. First, the delay incurred by invalid cues was reduced for cues on the high-frequency compared with the low-frequency cue positions (496 vs. 513 ms), *t*(15) = 5.69, *p* < .001, Cohen’s *d*_*z*_ = 1.42. Second, RTs to targets on the high-frequency cue position were delayed compared with targets on a low-frequency cue position (530 vs. 513 ms), *t*(15) = 2.57, *p* = .021, Cohen’s *d*_*z*_ = 0.64. Finally, there was no difference between valid cues on high-frequency and low-frequency cue positions (451 vs. 448 ms), *p* = .487. The same comparisons were also performed on error rates, but no significant results were observed, *p*s > .211.

Contrary to our predictions, analyses of the unbiased block showed no transfer of statistical learning. Neither the first nor the second comparison involving invalid trials was significant, neither in RTs nor in error rates, *p*s > .12. Unexpectedly, the comparison of valid conditions showed that RTs were shorter for valid cues on the high-frequency than the low-frequency cue positions (422 vs. 437 ms), *t*(15) = 4.56, *p* < .001, Cohen’s *d*_*z*_ = 1.14, which requires further research.

To better understand the transition from biased to unbiased trial blocks, we divided the 480 trials from the unbiased block into three blocks of 160 trials. Unfortunately, it was not possible to have smaller blocks, because the number of trials per condition was already very low. For the invalid conditions shown in Table 1, the trial numbers in the 160-trial blocks were reduced to 84, 24, and 12, respectively. For the valid conditions, the number of trials were reduced to 28 and 12, respectively. We calculated the differences of interest for each of the three 160-trial blocks and entered the difference values into a one-way analysis of variance (ANOVA), but found no effects, *p*s > .14. Possibly, the low number of trials reduced the power of the analysis, but more likely, the trials of interest were too infrequent to reliably trace the time course. Apart from the invalid condition where the cue appeared on the high-frequency position and the target on a low-frequency cue position (52.5% of trials), the conditions occurred only between 7.5% and 17.5% of trials. Therefore, the critical trials may have been too rare to reflect the transition from biased to unbiased processing. Nonetheless, we may conclude that the transition was rapid and occurred in fewer than 160 trials.

### Discussion

We evaluated whether the attentional suppression of the frequent cue position persisted in a block of trials with balanced probabilities. We found no transfer from biased to unbiased trial blocks, similar to some research on the additional singleton paradigm (Ferrante et al., 2018). Therefore, we conclude that suppression resulting from the frequent presentation of the cue at one location is short lived and subsides rapidly. However, we are unable to provide a more precise assessment of the time course because of limitations imposed by the experimental design.

## General discussion

We investigated attentional suppression with target-matching distractors. Previous studies on this topic yielded inconclusive evidence. One study argued for attentional suppression (Leber et al., 2016), but lacked a baseline condition in the RT task. Two others showed no evidence for suppression, but some evidence for the reduction of attentional capture by invalid cues (Ruthruff & Gaspelin, 2018; but see Burnham, 2019). We sought independent evidence for attentional suppression in cue–target paradigms by investigating cue and target processing at a location where the cue was frequently presented. Only target-matching cues showed evidence for attentional suppression of the frequent cue location, suggesting that feature-based attention guided attentional suppression. Attentional suppression had two effects: capture by invalid cues was reduced and target processing was impaired. These results show that participants did not only learn to better ignore salient distractors on the high-frequency cue location but suppressed stimulus processing on this location. Compared with previous studies using cue–target paradigms, the statistical learning procedure allowed for the evaluation of cue and target processing at the suppressed location. In contrast, previous investigations could not evaluate target processing because the target stimulus was never shown on the ignored locations (Burnham, 2018; Ruthruff & Gaspelin, 2018) or the respective analysis was not performed (Leber et al., 2016).

One open question concerns the valid conditions where cue-related and target-related processes were confounded. We did not have specific predictions about how these processes interact. The most straightforward prediction would be that reduced attentional capture and impaired target processing add up, which would predict longer RTs for valid cues on high-frequency than low-frequency cue positions. However, we observed no difference between valid conditions. Possibly, the presentation of cue and target on the same location created an intact object file (Carmel & Lamy, 2014, 2015) and the benefits of object continuity prevailed over attentional suppression. Further research is necessary to clarify this issue.

### Fixed versus variable locations in explicit procedures

Overall, the current study supports the previous conclusion of Leber et al. (2016) that statistical learning may lead to attentional suppression in cue–target paradigms. Further, our study is consistent with Ruthruff and Gaspelin’s (2018) observation that the delay incurred by invalid cues is reduced at ignored locations. In contrast, our results are at odds with Burnham (2018), who found no reduction of the delay at ignored locations. The discrepancy may result from the different procedures used to induce suppression. Ruthruff and Gaspelin (2018) used an explicit procedure and the to-be-ignored locations were fixed. In Burnham (2018), the to-be-ignored location was also explicit, but changed from trial to trial. Previous research has demonstrated that participants find it difficult not to pay attention to a color they are expected to ignore (Moher & Egeth, 2012) unless the color is fixed and participants are given many trials of practice (Cunningham & Egeth, 2016). Therefore, the difference between fixed and variable locations may explain the discrepancy between the study of Ruthruff and Gaspelin (2018) and the study of Burnham (2018). In a similar vein, Wang and Theeuwes (2018a) found no reduction of attentional capture in the additional singleton paradigm when the variable location of the color distractor was explicitly cued by an arrow (see also Heuer & Schubö, 2020). Nonetheless, there are instances where explicit cueing procedures with variable locations were effective. For instance, interference was reduced (Chao, 2010; Munneke, Van der Stigchel, & Theeuwes, 2008) or eliminated (Theeuwes, 1991; Yantis & Johnston, 1990) when arrows pointed to the location of an abrupt-onset distractor. Thus, there is consistent evidence for participant’s ability to explicitly ignore or suppress fixed locations, but the evidence for the suppression of variable locations is mixed. In any case, the learning effects in the current study break through the dichotomy of bottom-up and top-down control (Awh et al., 2012; Theeuwes, 2019), which is an interesting finding in a paradigm that is often described as a prime example of top-down control (Burnham, 2007; Büsel, Voracek, & Ansorge, 2018; Lamy, Leber, & Egeth, 2012; Schoeberl, Goller, & Ansorge, 2019; York & Becker, 2020).

### Spatial versus feature frequency

The effects of spatial frequency in our study differ markedly from a previous manipulation of feature frequency in the contingent capture paradigm. In search with two target colors, Berggren and Eimer (2019) observed that cueing effects were not affected by whether the cue appeared in the frequent or infrequent target color, suggesting that feature-based attention only reflects which color is task relevant, but ignores feature frequencies (but see Cosman & Vecera, 2014). In contrast, we find that the frequency of cue positions does affect cueing effects. The easiest explanation would be to assume that feature-based attention lacks sensitivity to feature frequency. That is, feature-based attention may be generated for all attentional templates alike. In contrast, location-based attention may be tuned to statistical regularities concerning the location of distractors. However, the dissociation between feature and spatial frequencies was not observed in recent experiments using the contingent capture paradigm. Stilwell, Bahle, and Vecera (2019) demonstrated that interference from a frequent distractor color was reduced compared with a less frequent color. In addition, suppression of the frequent-distractor position was more efficient when the distractor feature at this location remained the same (Failing, Feldmann-Wüstefeld, Wang, Olivers, & Theeuwes, 2019). These findings suggest that the frequency of distractor features, not only distractor position, modulates attentional capture. The reason for the discrepancy between results from the additional singleton and contingent capture paradigms may be that distractors in the additional singleton paradigm never matched the target features, whereas cues in the relevant studies on the contingent capture paradigm were target matching. Thus, effects of feature frequency in the additional singleton paradigm may be related to the stronger capture by novel events (Vatterott & Vecera, 2012; Zehetleitner, Goschy, & Müller, 2012), whereas the lack of effects of feature frequency in the contingent capture paradigm may reflect the need to establish feature-based attention for all attentional templates.

### The role of feature-based attention

Thus, feature-based attention may have played a much more prominent role in studies using the contingent capture paradigm than in studies using the additional singleton paradigm. Importantly, attentional capture in the additional singleton paradigm is brought about by the saliency of the distractors, not because feature-based attention was directed at the distractor feature. Thus, prominent explanations of attentional capture in the additional singleton paradigm rely on bottom-up control of attention mediated by the saliency map, which combines local contrast on individual feature maps (Fecteau & Munoz, 2006; Itti & Koch, 2001; Ptak, 2012). The theory of dimensional weighting further assumes that the feature maps pertaining to the task-relevant stimulus dimension are given more weight in the overall saliency computation (Found & Müller, 1996; Liesefeld, Liesefeld, Pollmann, & Müller, 2019). Sauter et al. (2019) noted that effects of distractor frequency were much larger if the distractor was defined on the same dimension as the target. In addition, target processing at the frequent-distractor location was impaired for same-dimension, but not for different-dimension distractors. However, other studies reported impaired target processing with different-dimension distractors (Failing, Wang, & Theeuwes, 2019; Wang & Theeuwes, 2018b, c; Wang, van Driel, Ort, & Theeuwes, 2019). The discrepancy was resolved by showing that impaired target processing with distractors from a different dimension was only reliable with trial-wise color swaps between target and distractor (Allenmark, Zhang, Liesefeld, Shi, & Müller, 2019; B. Zhang, Allenmark, Liesefeld, Shi, & Müller, 2019), possibly because distractor suppression is generally less successful with random feature changes (Graves & Egeth, 2016; Kerzel & Barras, 2016). In the current study, there was impaired target processing despite a fixed target feature, showing that results from the additional singleton paradigm and the contingent capture paradigm overlap to some degree, but significant differences remain.

In sum, the present study provides evidence for attentional suppression in the contingent capture paradigm. We manipulated the frequency of cue locations and found that invalid cues captured attention less on the frequent cue location. At the same time, target processing was impaired on this location. Because statistical learning only occurred with target-matching cues, we suggest that feature-based attention guided attentional suppression, just as it guides attentional enhancement.

## Footnotes

1 Leber et al. (2016) presented perceptual probes on 20% of the trials in their Experiment 2. They found perceptual performance (accuracy) to be worse at the expected distractor location than at other locations, which suggests that suppression occurred. However, the large effect of endogenous cues on speeded RTs in Experiment 1 was not found in the perceptual measure of Experiment 2. Therefore, it is unclear whether the two dependent variables reflect the same underlying process.
